# Full-length transcriptome analysis revealed that 2,4-dichlorophenoxyacetic acid promoted *in vitro* bulblet initiation in lily by affecting carbohydrate metabolism and auxin signaling

**DOI:** 10.3389/fpls.2023.1236315

**Published:** 2023-09-20

**Authors:** Cong Gao, Lin Zhang, Yunchen Xu, Yue Liu, Xiao Xiao, Liu Cui, Yiping Xia, Yun Wu, Ziming Ren

**Affiliations:** ^1^ Genomics and Genetic Engineering Laboratory of Ornamental Plants, College of Agriculture and Biotechnology, Zhejiang University, Hangzhou, Zhejiang, China; ^2^ Laboratory of Flower Bulbs, Department of Landscape Architecture, Zhejiang Sci-Tech University, Hangzhou, Zhejiang, China

**Keywords:** starch synthesis and degradation, sucrose unloading, auxin signaling, full-length sequencing, *Lilium*

## Abstract

Bulblet initiation, including adventitious bud initiation and bulblet formation, is a crucial process for lily and other bulbous flowers that are commercially propagated by vegetative means. Here, by a hybrid strategy combining Pacific Biosciences (PacBio) full-length sequencing and Illumina RNA sequencing (RNA-seq), high-quality transcripts of *L. brownii* (*Lb*) and its variety, *L. brownii* var. giganteum (*Lbg*), during *in vitro* bulblet initiation were obtained. A total of 53,576 and 65,050 high-quality non-redundant full-length transcripts of *Lbg* and *Lb* were generated, respectively. Morphological observation showed that *Lbg* possessed a stronger capacity to generate bulblets *in vitro* than *Lb*, and 1 mg L^−1^ 2,4-dichlorophenoxyacetic acid (2,4-D) significantly increased bulblet regeneration rate in two lilies. Screening of differentially expressed transcripts (DETs) between different stages and Mfuzz analysis showed 0 DAT to 1 DAT was the crucial stage with the most complex transcriptional change, with carbohydrate metabolism pathway was significantly enriched. In addition, 6,218 and 8,965 DETs were screened between the 2,4-D-treated group and the control group in *Lbg* and *Lb*, respectively. 2,4-D application had evident effects on the expression of genes involved in auxin signaling pathway, such as TIRs, ARFs, Aux/IAAs, GH3s and SAURs. Then, we compared the expression profiles of crucial genes of carbohydrate metabolism between different stages and different treatments. SUSs, SUTs, TPSs, AGPLs, GBSSs and SSs showed significant responses during bulblet initiation. The expression of CWINs, SUTs and SWEETs were significantly upregulated by 2,4-D in two lilies. In addition, 2,4-D increased the expression of starch degradation genes (AMYs and BAMs) and inhibited starch synthesis genes (AGPLs, GBSSs and SSs). SBEs were significantly upregulated in *Lbg* but not in *Lb*. Significant co-expression was showed between genes involved in carbohydrate metabolism and auxin signaling, together with transcription factors such as bHLHs, MYBs, ERFs and C3Hs. This study indicates the coordinate regulation of bulblet initiation by carbohydrate metabolism and auxin signaling, serving as a basis for further studies on the molecular mechanism of bulblet initiation in lily and other bulbous flowers.

## 1 Introduction

Bulbous flowers, which are highly popular in the world floriculture market, are usually commercially propagated by vegetative means, especially by bulbs, to maintain phenotypic uniformity and genetic purity. Bulblet initiation, including the process of adventitious/axillary bud initiation and bulblet formation, has been reported in multiple bulbous plants from plant tissues *in vitro* (Van Aartrijk and Blom-Barnhoorn, 1984; Li et al., 2014; Ren et al., 2017; Lv et al., 2020). Lily (*Lilium* spp.), a perennial monocotyledon of the family Liliaceae, is one of the major bulbous crops in the floriculture industry with high ornamental, medical and edible value (Lee et al., 2013; Xu et al., 2017; Wu et al., 2019). Due to its strong bulblet formation capacity *in vitro*, lily is considered as an appropriate experimental material for the study of bulblet initiation and its underlying mechanisms. Studies on bulblet initiation in lily have focused mainly on the influence of wounds, temperature treatment and exogenous phytohormones (Van Aartrijk and Blom-Barnhoorn, 1984), changes in endogenous carbohydrate and hormone contents, and the expression level of genes involved in carbohydrate and phytohormone metabolism (Li et al., 2014; Wu et al., 2020).

Carbohydrate metabolism plays a vital role in bulblet initiation of multiple bulbous flowers. Therein, starch metabolism has been reported to be ubiquitously involved. Storage starch in mother scales could act as a carbon source for bulblet initiation. At the bulblet appearance and enlargement stage, the enzymes involved in the starch synthetic direction, such as ADP-glucose Pyrophosphorylase (AGPase, EC 2.7.7.27), Starch Synthase (SS, EC 2.4.1.21), Starch Branching Enzyme (SBE, EC 2.4.1.18) and Granule-bound Starch Synthase (GBSS, EC 2.4.1.242), showed a decreasing trend in mother scales but higher gene expression levels in newly formed bulblets, while the enzyme in the starch cleavage direction, Starch Debranching Enzyme (DBE, EC 3.2.1.10), showed higher expression levels in scales than in bulblets in lily (Li et al., 2014). Similarly, in *Lycoris*, soluble sugars derived from starch degradation in the outer scales were transported into the inner scales and promote bulblet initiation and development through starch synthesis, especially through AGPases (Xu et al., 2020a). Sucrose, the main form of transported sugar in higher plants, were also considered to regulate bulblet initiation. Sucrose Synthase (SUS, EC 2.4.1.13) and Invertase (INV, EC 3.2.1.26, including Cell Wall Invertase (CWIN), Vacuolar Invertase (VIN) and Cytoplasmic Invertase (CIN)), mainly hydrolyzing sucrose, presented higher expression levels in mother scales and bulblets at stages of bulblet appearance and enlargement in lily (Li et al., 2014). A clear shift was observed from CWIN-catalyzed to SUS-catalyzed sucrose cleavage patterns, meanwhile, sucrose unloading pathway changed from apoplasmically to symplasimically at the key shoot-to-bulblet transition stage in *Lilium* Oriental Hybrids ‘Sorbonne’ (Wu et al., 2021). Similarly, *CWIN* and *SUS* exhibited exactly opposite expression patterns during the competence stage of bulblet regeneration in *Lycoris* (Ren et al., 2021). The above results indicated that the transition from bud initiation to bulblet enlargement was usually accompanied by the change of dominant sucrose unloading pathway.

Auxin, a key phytohormone, regulates diverse aspects of plant growth and developmental processes through its dynamic differential distribution (Vanneste and Friml, 2009). The biosynthesis of indole-3-acetic acid (IAA), the main naturally occurring auxin, in higher plants requires two steps: first, tryptophan is converted to indole-3-pyruvate (IPA) by Tryptophan Aminotransferase (TAA) or Tryptophan Aminotransferase Related (TAR); second, IAA is produced from IPA by YUC family (YUC) (Zhao, 2012). A previous study revealed that adventitious bulblets of lily were formed at the basal edge of the explant under tissue culture conditions, which caused by basipetal auxin transport (Van Aartrijk and Blom-Barnhoorn, 1984). Different auxin concentrations showed different effects on the process of bulblet formation. Auxin likely promoted the initiation of bulbils and then inhibited further bulbil formation in lily (Yang et al., 2017). In *Lycoris*, endogenous IAA content showed an increase and then a decrease during bulblet initiation and development, which were consistent with the expression patterns of genes involved in IAA synthesis and signal transduction (Xu et al., 2020a).

To obtain more genetic information, Illumina next-generation sequencing (NGS) technology has been widely used in the study of the process of bulblet formation in multiple bulbous plants, including *Lilium* (Li et al., 2014; Yang et al., 2017), *Lycoris* (Ren et al., 2022) and sweet potato (*Ipomoea batatas*) (Firon et al., 2013). In *Lilium*, transcriptome analysis was used to elucidate the molecular mechanism of bulblet/bulbil formation and development. (Li et al., 2014; Yang et al., 2017). However, the early stage of bulblet formation in *Lilium* and even in bulbous flowers remains unclear. Recently, the single-molecule real-time (SMRT) sequencing technology of the PacBio system has offered a new third-generation sequencing platform, which possesses advantages such as long read lengths (length > 10 kb), high consensus accuracy and a low degree of bias (Hu et al., 2022), which is an available and reliable strategy to generate more accurate and comprehensive genetic information. A recent study obtained *Lilium* Oriental Hybrids ‘Sorbonne’ transcriptome during induction of aerial bulbil using the combination of SMRT and NGS technology (Li et al., 2022).

Here, we explore the *in vitro* bulblet initiation process of *Lilium brownii* (*Lb*) and *Lilium brownii* var. *giganteum* (*Lbg*), a variant of *Lb* (Li et al., 2007), through careful morphological observation, and then divided the process into four stages. Then, a hybrid strategy combining Pacific Biosciences (PacBio) full-length sequencing and Illumina sequencing was conducted. Through differentially expression transcript (DET) screening and Mfuzz analysis, we identified key metabolic pathways and candidate genes during bulblet initiation. In addition, DET screening was also performed between the 2,4-dichlorophenoxyacetic acid (2,4-D)-treated group and the control group, to explore how 2,4-D, a synthetic auxin analog, affect the process of bulblet initiation. Furthermore, we hypothesized that carbohydrate metabolism and auxin signaling coordinately regulate bulblet initiation, with several transcriptional factors (TFs) involved in the regulation of this process. Our findings provide a comprehensive understanding of the molecular mechanism underlying the process of bulblet initiation in lily.

## 2 Materials and methods

### 2.1 Plant materials and growth conditions

Bulblet induction experiments were conducted at the Physiology & Molecular Biology Laboratory of Ornamental Plants and Tissue Culture Laboratory of Ornamental Plants at Zhejiang University, Hangzhou (118°21’-120°30’E, 29°11′-30°33’N), China. *In vitro* seedlings of *Lb* and *Lbg* were cultured at 25 ± 2°C under a 12:12 h light:dark photoperiod with 60 μmol photons m^-2^ s^-1^. Healthy outer scales without damage were removed carefully from fresh *in vitro* bulbs (4~6 cm in circumference) and then cultured for 14 days on basal Murashige and Skoog (MS) medium (Murashige and Skoog, 1962) containing 6% sucrose and 0.3% Phytagel (P8169, Sigma-Aldrich, St. Louis, MO, USA) (pH 5.8), to which was added 0 mg L^−1^ or 1 mg L^−1^ 2,4-D, with the adaxial side facing upward. Each treatment contained three biological replicates, and each replicate included 120 scales.

### 2.2 Morphological and histological observation

Morphological changes of bulblet initiation in *Lb* and *Lbg* were observed under a stereomicroscope (SZM745T, OPLENIC, China). The regeneration rate and propagation efficiency of bulblets were calculated as follows: Regeneration rate = number of scales that produced adventitious buds/total number of scales; Propagation efficiency = total number of produced buds/total number of scales. Representative data were supported by three biological replicates, each containing 120 repeats. Transverse sections of scales at the proximal end where adventitious buds initiated were stained with periodic acid-Schiff (PAS) and Naphthol Yellow S as previously described (Ren et al., 2017), and then observed using an upright light microscope (Eclipse E100, Nikon, Japan).

### 2.3 Sample collection

The bulblet initiation process of *Lb* and *Lbg* was divided into four crucial stages according to the results of morphological and histological observations: stage of scale detachment (0 DAT; DAT, days after the treatment of detaching the scale from the basal plate), stage of wounding response and early regeneration competence (1 DAT), stage of adventitious bud initiation (8 DAT) and stage of adventitious bud swelling and bulblet formation (14 DAT). The entire scales used for bulblet induction at 0 DAT, 1 DAT, 8 DAT and 14 DAT were sampled, frozen in liquid nitrogen and stored at -80°C for total RNA extraction. Sampling was performed with three biological replicates for each stage.

### 2.4 Generation of the full-length reference transcripts for *Lb* and *Lbg*

Total RNA of scale samples at four stages of *Lb* and *Lbg* cultured on 2,4-D-free (0 mg L^-1^ 2,4-D) and 2,4-D-containing (1 mg L^-1^ 2,4-D) medium was extracted using an EASYspin Plus Complex RNA Kit (RN53, Aidlab Bio, China) according to the manufacturer’s instructions. Total RNA from each sample was equally mixed to generate a pool and then synthesized to first-strand cDNA using Clontech SMARTer PCR cDNA Synthesis Kit (Clontech, Mountain View, CA, USA). Large-scale PCR was performed using the BluePippin™ Size Selection System (Sage Science, Beverly, MA, USA). The SMRTbell template libraries were constructed and then sequenced on the PacBio Sequel platform. The following methods for generating full-length reference transcripts referenced Li et al. (2022) with some modifications. The high-quality full-length transcripts were removed rebundancy using CD-HIT v4.6.142 (Li and Godzik, 2006). BUSCO 5.2.0 was used to assess the quality and completeness of the reference transcripts using the official BUSCO datasets (liliopsida_odb10) (Manni et al., 2021).

Total RNA of scale samples was sequenced with the Illumina Xten 4000 platform (Illumina, San Diego, CA, USA). Quality control (QC) for each Illumina transcriptome was performed by fastp (v0.19.7) with default parameters (Chen et al., 2018). Clean reads were separately mapped to their corresponding reference transcripts by Bowtie2 (v2.3.4), and the expression levels of each transcript, including reads count and fragments per kilobase million (FPKM), were calculated by RSEM (v1.3.1) (Li and Dewey, 2011; Langmead and Salzberg, 2012).

### 2.5 Transcript annotation

Each transcript was annotated by the eggNOG-mapper webserver (http://eggnog-mapper.embl.de/) with an e-value of ≤ 1e^-5^ and identity of ≥ 60% with Viridiplantae (green plants) selected as the taxonomic scope (Huerta-Cepas et al., 2019; Cantalapiedra et al., 2021). As a result, transcripts were functionally annotated based on the following databases: Pfam, NCBI nonabundant (NR), Kyoto Encyclopedia of Genes and Genomes (KEGG) and Gene Ontology (GO). The PlantTFDB v5.0 database (Jin et al., 2017) was used to predict TFs and their homologous genes in *Arabidopsis thaliana* of the full-length reference transcripts in *Lb* and *Lbg*. Venn diagrams were generated using Evenn (http://www.ehbio.com/test/venn/#/, Chen et al., 2021).

### 2.6 DET screening and enrichment analysis

Differential expression analysis was performed using R package DESeq2 (v1.32.0) (Love et al., 2014). Transcripts with a false discovery rate (FDR) ≤ 0.05 and a threshold of |log2-fold changes| ≥ 2 were recognized as DETs. Each group of DETs was calculated for KEGG pathways by clusterProfiler (v4.0.5) using an p-value of 0.05 to find significant enrichments (Wu et al., 2021). Time-series cluster analysis was performed by the Mfuzz package (v2.50.0) in R software, with the low expression level transcripts with FPKM ≤ 5 removed.

### 2.7 Quantitative real-time PCR validation

To confirm the results of the transcript expression levels from RNA-Seq, six DETs of *Lbg* and six DETs of *Lb* were selected for expression analysis using quantitative real-time PCR (qRT-PCR). Total RNA (1 μg) of each sample was reverse transcribed by the PrimeScript™ RT reagent Kit with gDNA Eraser (RR047A, TaKaRa, Dalian, China). The diluted (1:30) cDNA was used as the template for qRT-PCR analysis. Gene-specific primers were designed by the NCBI Primer-BLAST tool (Ye et al., 2012) and are listed in **Supplementary Table S2**. Then, qRT-PCR was performed with TB Green™ Premix Ex Taq™ Kit (RR420A, TaKaRa, Dalian, China) in a Bio-Rad Connect™ Optics Module (Bio-Rad, CA, USA). All reactions were conducted in triplicate, and the 2^–ΔΔCT^ method was applied to calculate the relative expression level using *GAPDH* as the reference gene.

### 2.8 Nonstructural carbohydrate content assay

Total starch contents of scales at different stages were measured using a Starch Content Kit (A148-1-1, Nanjing Jiancheng Bioengineering Institute, Nanjing, China) following the manufacturer’s protocol. Three replicates were included in each assay. Sucrose, fructose, and glucose contents were measured by high-performance liquid chromatography (HPLC) (e2695, Waters, MA, USA) equipped with Refractive Index (RI) Detector 2414 (Waters) according to the previous method in Liu et al. (2020).

### 2.9 Determination of IAA concentration

IAA extraction and quantification were performed using a previously described method with slight modifications (Guo et al., 2016). Briefly, frozen scale sample (100 mg) of each treatment at each stage was weighed in a 10-mL centrifuge tube, and homogenized in 1 mL of ethyl acetate that had been spiked with D5-IAA (C/D/N Isotopes) as an internal standard at a final concentration of 100 ng mL^−1^. The tubes were centrifuged at 12 000 rpm for 10 min at 4°C. The resulting supernatant was dried by blowing under N2. The residue was resuspended in 0.5 mL of 70% (v/v) methanol and centrifuged, and the supernatants were then analyzed in a triple quadrupole mass spectrometer (6470, Agilent Technologies, CA, USA).

### 2.10 Statistical analysis

One-way analysis of variance (ANOVA) was used to compare differences among different indices or treatments via SPSS 26.0 (IBM Corp., Armonk, NY, USA). Correlation analyses between gene expression data in *Lb* and *Lbg* were performed using Pearson’s two-tailed tests and visualized by Cytoscape v3.7.1 (Shannon et al., 2003).

## 3 Results

### 3.1 Stage division according to morphological and histological observation

**Figure 1:**
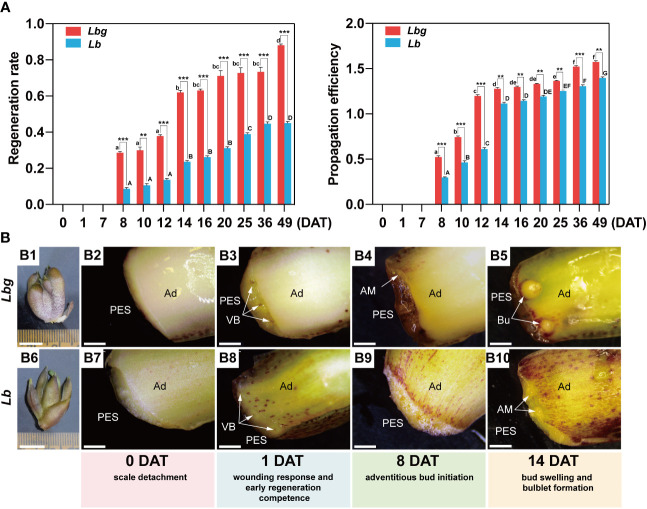
Morphological observation during *in vitro* bulblet initiation in *Lbg* and *Lb.* **(A)** Regeneration rate and propagation efficiency during *in vitro* bulblet initiation. Regeneration rate, number of scales that produced adventitious buds/total number of scales. Propagation efficiency, total number of produced buds/total number of scales. Lowercase and uppercase letters represent significant differences (*p* < 0.001) for relevant parameters within *Lbg* and *Lb*, respectively. Asterisks indicate significant differences for relevant parameters between *Lbg* and *Lb* (**Differences significant at *p* < 0.01; ***Differences significant at *p* < 0.001). Representative data were supported by three biological replicates containing 120 repeats each. **(B)** *In vitro* bulb of which scales were used for bulblet induction **(B1, B6)** and key stages during bulblet initiation in *Lbg* **(B2-B5)** and *Lb* **(B7-B10)**. PES, proximal end of scale; Ad, adaxial side of scale; VB, vascular bundle; AM, adventitious meristem; Bu, bulblet. The white arrows represent vascular bundles (B3 and B4), adventitious meristems (B4 and B10) or bulblets (B9). Bars, 1 cm **(B1, B6)** and 1 mm **(B2-B5, B7-B10)**.

Throughout the bulblet initiation and development process (0 DAT to 49 DAT), the regeneration rate and propagation efficiency were calculated. It is showed that visible adventitious buds occurred at 8 DAT, and the number of newly formed bulblets was significantly increased until 14 DAT and then tended to plateau until 25 DAT or later in both *Lbg* and *Lb* (**Figure 1A**). In addition, 1 DAT was considered as the crutial wounding response stage according to our previous studies. Based on morphological observations, browning substances began to accumulate in the transverse section at 1 DAT (**Figures 1B3, B8**). At 8 DAT, adventitious buds formed at the adaxial side rather than the abaxial side of the scale, mainly around lateral vascular bundles (**Figures 1B4, B9**; **Supplementary Figures S1B, E**). Then, adventitious buds swelled, and visible bulblets occurred at 14 DAT (**Figures 1B5, B10**; **Supplementary Figures S1C, F**). Taken together, we divided the early bulblet initiation process into four stages: 0 DAT (scale detachment), 1 DAT (wounding response and early regeneration competence), 8 DAT (adventitious bud initiation) and 14 DAT (bud swelling and bulblet formation). Moreover, the bulblet regeneration rate and propagation efficiency of *Lbg* were both significantly higher than those of *Lb* from 8 DAT to 49 DAT (**Figure 1A**). At 14 DAT, the regeneration rate and propagation efficiency of *Lbg* were 0.619 and 1.677, respectively, significantly higher (*p* < 0.001 and *p* < 0.01, respectively) than those in *Lb* (0.236 and 1.114, respectively) (**Figure 1A**). The above results indicated that *Lbg* possesses a stronger capacity to generate bulblets *in vitro* than *Lb*.

### 3.2 Quality assessment of obtained reference transcripts

**Figure 2:**
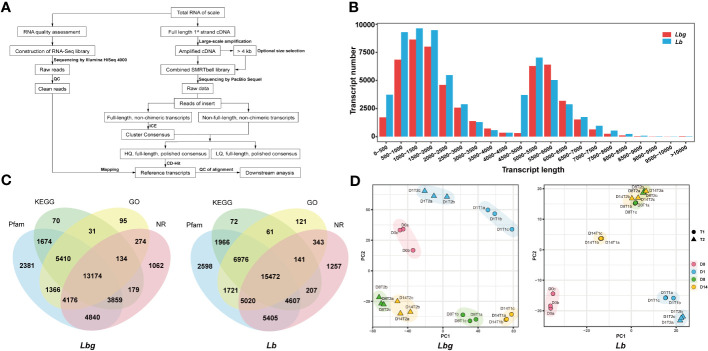
Reference transcripts generation of lily samples. **(A)** Workflow of lily sample sequencing. **(B)** Distribution of the length of reference transcripts. **(C)** Functional annotation of reference transcripts by Pfam, KEGG, GO and Nr databases. **(D)** Principal component analysis (PCA) plot of the samples. T1 and T2 represent samples of the control group and 2,4-D-treated group, respectively. D0, D1, D8 and D14 represent samples of 0 DAT, 1 DAT, 8 DAT and 14 DAT, respectively. The letters (a-c) represent three biological replicates.

Full-length transcriptome sequencing was conducted to generate complete and accurate gene information during bulblet initiation (**Figure 2A**). The reference transcripts for *Lbg* were first obtained with 53,576 nonredundant transcripts of an average length of 3,108 bp, and 80.79% of the *Lbg* clean reads obtained by Illumina RNA-Seq mapped to the *Lbg* reference transcripts (**Supplementary Table S1**). Similarly, the *Lb* full-length reference transcripts were obtained, consisting of 65,050 nonredundant transcripts of an average length of 2,965 bp, with 81.85% *Lb* clean reads mapped to them (**Supplementary Table S1**). Moreover, the *Lbg* and *Lb* reference transcripts had 73.1% and 75.9% of the conserved plant genes by BUSCO 5.2.0, respectively (**Supplementary Table S1**). These results confirmed the reliability of these two transcriptomes for downstream analysis. The length of transcripts ranged from 294 bp to 13,738 bp in *Lbg* and from 292 bp to 14,486 bp in *Lb*, with a median length of 2,139 and 2,021 bp and a mean length of 3,108 and 2,965 bp in *Lbg* and *Lb*, respectively (**Figure 2B**). Moreover, a total of 38,725 (72.3%) and 45,967 (70.7%) transcripts were annotated to the NR, GO, KEGG and Pfam databases in *Lbg* and *Lb*, respectively (**Figure 2C**). Among them, 13,174 and 15,472 transcripts were annotated in all the four databases.

Principal component analysis (PCA) showed that there were obvious differences of expression patterns among different stages under the same treatment conditions, except for 8 DAT and 14 DAT in *Lb* in the medium supplemented with 1 mg L^-1^ 2,4-D (**Figure 2D**). In addition, a large deviation was also observed between the 2,4-D treatment group and the control group at the same stage. The results of sample clustering were consistent with the PCA results (**Supplementary Figure S2**). The above results further validated that obvious differences existed among stages during bulblet initiation, and 2,4-D influenced this process in both *Lbg* and *Lb*.

Six transcripts of *Lbg* (Isoform 22016, 21863, 28761, 46833, 29525 and 20315) and six transcripts of *Lb* (Isoform 31916, 25438, 39080, 36307, 34089 and 27602) were randomly selected for qRT-PCR to validate the differential expression by RNA-Seq. The results showed that the differential expression levels of these selected transcripts by qRT-PCR were highly consistent with those obtained by RNA-Seq (**Supplementary Figure S3**), confirming the reliability and accuracy of the RNA-Seq data.

### 3.3 Stage-specific DET screening and Mfuzz analysis revealed possible events of different stages

**Figure 3:**
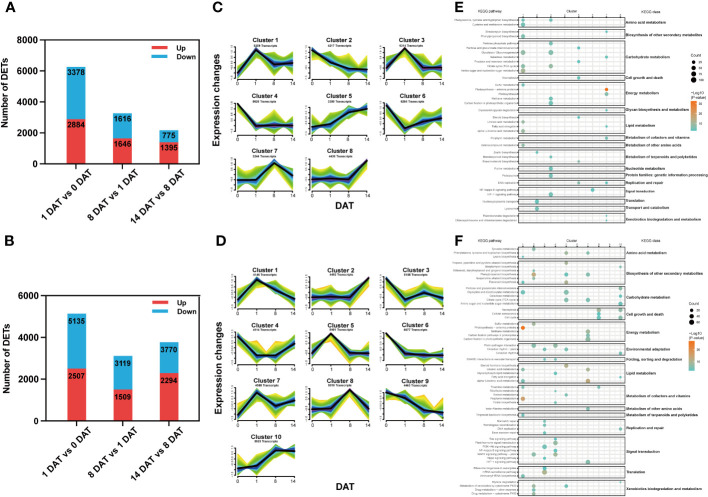
Stage-specific DETs screening and Mfuzz analysis of reference transcripts. **(A, B)** Number of DETs (|log2-fold changes| ≥ 2) identified between different stages in *Lbg* **(A)** and *Lb* **(B)**. **(C, D)** Stage-specific DETs were classified into eight clusters of *Lbg* **(C)** and ten clusters of *Lb* **(D)** through Mfuzz analysis. **(E, F)** Bubble charts of KEGG pathway enrichment analysis of each cluster in *Lbg* **(E)** and *Lb* **(F)**.

To identify transcriptional changes between distinct bulblet initiation stages, we compared the transcript expression profiles of adjacent stages in each lily, including 1 DAT versus (vs) 0 DAT (Group 1), 8 DAT vs 1 DAT (Group 2) and 14 DAT vs 8 DAT (Group 3). In total, 11,964 and 18,834 stage-specific DETs were screened in *Lbg* and *Lb*, respectively (**Figures 3A, B**). Clearly, Group 1 had the largest number of DETs among the three groups in both *Lbg* and *Lb* (**Figures 3A, B**), indicating that there were more complex transcriptional changes from 0 DAT to 1 DAT than in the following stages.

To define the temporal characteristics of the transcript dataset, we performed clustering analysis of 41,680 and 48,314 transcripts by Mfuzz in *Lbg* and *Lb*, respectively. The transcripts were divided into eight and ten clusters in *Lbg* and *Lb*, respectively (**Figures 3C, D**), and KEGG pathway enrichment analysis of these clusters was conducted (**Figures 3E, F**). The transcripts of clusters 1 and 3 in *Lbg* and clusters 5 and 7 in *Lb* could be candidates for the early response to scale detachment, since the highest expression of these clusters was exhibited at 1 DAT. The pathway “citrate cycle (TCA cycle)” (map00020), which is involved in carbohydrate metabolism, was upregulated in all the four candidate clusters (**Figures 3E, F**). The transcripts of cluster 7 in *Lbg* and cluster 8 in *Lb* were highly expressed at 8 DAT, with “DNA replication” (map03030) and “glycosaminoglycan degradation” (map00531) enriched in cluster 7 (*Lbg*), and “cell cycle” (map04110), “cellular senescence” (map04218) and “necroptosis” (map04217) enriched in cluster 8 (*Lb*), indicating that this stage could be associated with cell growth and death (**Figures 3E, F**). Moreover, the expression levels of transcripts in cluster 8 (*Lbg*) and cluster 2 (*Lb*) were increased at 14 DAT, and transcripts in cluster 2 (*Lb*) were mainly enriched in “biosynthesis of other secondary metabolites” and “xenobiotics biodegradation and metabolism” classes, which might play important functional roles in the bulblet swelling stage (**Figures 3E, F**).

### 3.4 2,4-D treatment promoted the process of *in vitro* bulblet initiation

**Figure 4:**
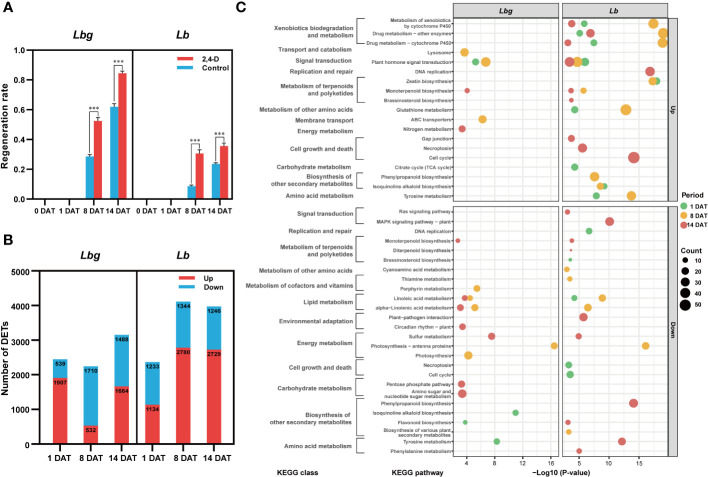
2,4-D-related DETs screening between the control group and 1 mg L^-1^ 2,4-D-treated group of reference transcripts. **(A)** Regeneration rate of the control group and 2,4-D-treated group during *in vitro* bulblet initiation. Regeneration rate = number of scales that produced adventitious buds/total number of scales. ***Differences significant at *p* < 0.001. **(B)** Number of DETs (|log2-fold changes| ≥ 2) identified between the control group and 2,4-D-treated group. **(C)** Bubble charts of KEGG pathway enrichment analysis of 2,4-D-related DETs.

To explore the effect of the exogenous application of 2,4-D on *in vitro* bulblet initiation, 1 mg L^-1^ 2,4-D was added to the medium for bulblet induction. Results showed that the regeneration rate was significantly higher (*p* < 0.001) in 2,4-D-treated group than in the control group in both *Lbg* and in *Lb* (**Figure 4A**). More specifically, the 2,4-D treatment increased the regeneration rate by 1.84-fold in *Lbg* and 3.55-fold in *Lb* at 8 DAT, and 1.36-fold in *Lbg* and 1.51-fold in *Lb* at 14 DAT, indicating that the promotion effect of 2,4-D was stronger in *Lb*. Next, we screened 6,218 (3,175 upregulated, 3,043 downregulated) and 8,965 (5,382 upregulated, 3,583 downregulated) DETs between 2,4-D-treated group and the control group at 1 DAT, 8 DAT and 14 DAT in *Lbg* and *Lb*, respectively (**Figure 4B**). KEGG pathway enrichment analysis showed that, with 2,4-D treatment, “plant hormone signal transduction” (map04075) was upregulated in all the three stages in *Lb* and was also upregulated at 1 DAT and 8 DAT in *Lbg* (**Figure 4C**), indicating that 2,4-D could promote phytohormone responsiveness throughout the bulblet initiation process. Thus, 2,4-D had a promoting effect on *in vitro* bulblet initiation, and this effect might be closely related to phytohormone signal transduction.

### 3.5 Changes in auxin-related genes during bulblet initiation

**Figure 5:**
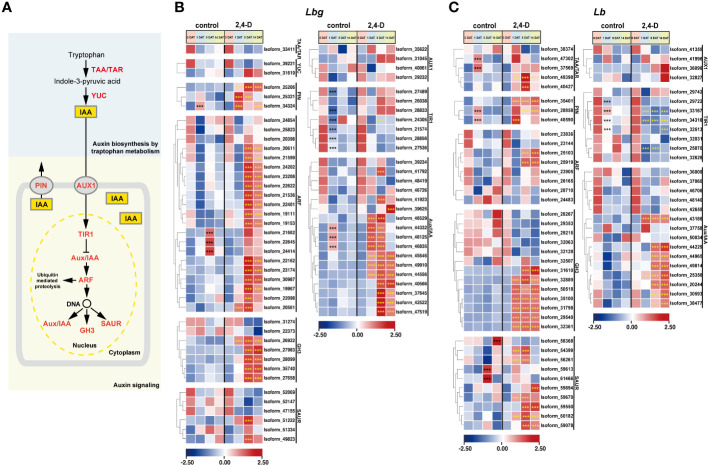
Expression patterns of stage-specific and 2,4-D related DETs involved in pathways of auxin biosynthesis and signaling. **(A)** Pathway of indole-3-acetic acid (IAA) biosynthesis and signaling. TAA, tryptophan aminotransferase; TAR, tryptophan aminotransferase related; YUC, YUC family; PIN, PIN-formed protein family; AUX1, AUX1/LAX symporters; TIR1, transporter inhibitor response 1, Aux/IAA, indole-3-acetic acid inducible; ARF, auxin response factor; GH3, GH3 family; SAUR, small auxin upregulated RNA. **(B, C)** Expression patterns of DETs involved in auxin biosynthesis and signaling in *Lbg* **(B)** and *Lb* **(C)**. ***Differences significant at *p* < 0.001. The black asterisk represents a significant difference compared to 0 DAT in the control group. The yellow asterisk represents a significant difference in the 2,4-D-treated group compared to the control group at the same stage.

Considering the effect of auxin on bulblet initiation in various bulbous flowers, and the “plant hormone signal transduction” pathway was in response to exogenous 2,4-D application, we focused on the expression patterns of all screened DETs involved in auxin biosynthesis and signaling (**Figure 5A**). It is obvious that all screed DETs encoding Transporter Inhibitor Response 1 (TIR1) (six in *Lbg* and four in *Lb*) were significantly downregulated (*p* < 0.001) at 1 DAT in both *Lbg* and *Lb* (**Figures 5B, C**). Particularly, in *Lb*, the downregulation of four TIR1s (Isoform 33167, 34316, 32513 and 25870) were significantly enhanced (*p* < 0.001) by 2,4-D treatment (**Figure 5C**). Besides, 2,4-D significantly promoted (*p* < 0.001) the expression of two Tryptophan Aminotransferase (TAA) and Tryptophan Aminotransferase Related (TAR) (Isoform 49388 and 40427) in *Lb*, which could promote the endogenous synthesis of auxin (**Figure 5C**). Correspondingly, the content of endogenous IAA in the scales was significantly higher (*p* < 0.05) under 2,4-D treatment than that of the control group at 8 DAT and 14 DAT in *Lb* (**Supplementary Figure S4**). These changes above may lead to a stronger increase of regeneration rate in *Lb* than in *Lbg*.

Three Auxin Response Factors (ARFs) of *Lbg* (Isoform 21662, 22645 and 24414) were significantly (*p* < 0.001) upregulated at 8 DAT in the control group, and 15 ARFs in *Lbg* and two ARFs in *Lb* were significantly (*p* < 0.001) upregulated at 8 DAT with 2,4-D treatment, following the downregulation of TIR1s (**Figures 5B, C**), indicating that 2,4-D could promote auxin signaling pathway during bulblet initiation. The significantly higher expression levels of DETs (*p* < 0.001) encoding some GH3 (five in *Lbg* and seven in *Lb*), Small Auxin Upregulated RNA (SAUR) (two in *Lbg* and seven in *Lb*) and Indole-3-acetic Acid Inducible (Aux/IAA) (14 in *Lbg* and eight in *Lb*) in the 2,4-D-treated group than in the control group at 1 DAT or 8 DAT further support the above idea (**Figures 5B, C**). Moreover, 2,4-D also promoted all screed DETs encoding PIN-formed protein family (PIN) (three in *Lbg* and three in *Lb*) after 1 DAT (**Figures 5B, C**). Overall, the promotion of auxin signaling pathway by 2,4-D application might contribute to the enhancement of bulblet regeneration ability.

### 3.6 Changes in key genes involved in sucrose and starch metabolism during bulblet initiation

**Figure 6:**
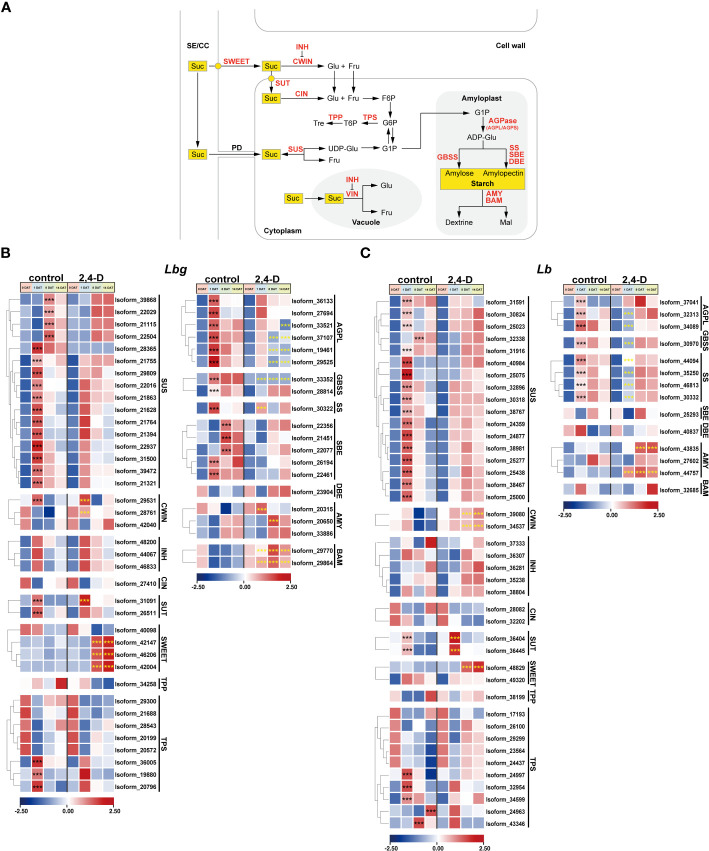
Expression patterns of stage-specific and 2,4-D related DETs involved in sucrose and starch metabolism pathway. **(A)** Sucrose and starch metabolism pathway. SE/CC, sieve element/companion cell complex; PD, plasmodesma; Suc, sucrose; Glu, glucose; Fru, fructose; Tre, trehalose; Mal, maltose; UDP-Glu, UDP-glucose; F6P, fructose-6-phosphate; G6P, glucose-6-phosphate; G1P, glucose-1-phosphate; ADP-Glu, ADP-Glucose; SWEET, SWEET sucrose-efflux transporter family; SUT, sucrose transporter; SUS, sucrose synthase; CWIN, cell wall invertase; VIN, vacuolar invertase; INH, invertase inhibitor; CIN, cytoplasmic invertase; TPP, trehalose 6-phosphate phosphatase; TPS, trehalose 6-phosphate synthase; AGPL/AGPS, large/small subunit of ADP-glucose pyrophosphorylase (AGPase); GBSS, granule-bound starch synthase; SS, starch synthase; SBE; starch branching enzyme; DBE, starch debranching enzyme; AMY, amylase, BAM, β-amylase. **(B, C)** Expression patterns of DETs involved in sucrose and starch metabolism in *Lbg* **(B)** and *Lb* **(C)**. ***Differences significant at *p* < 0.001. The black asterisk represents a significant difference compared to 0 DAT in the control group. The yellow asterisk represents a significant difference in the 2,4-D-treated group compared to the control group at the same stage.

Sucrose and starch metabolism was repeatedly reported to play an essential role in bulblet initiation in various bulbous flowers. Here, we focused on the expression patterns of key enzymes, transporters, and regulators involved in sucrose and starch metabolism pathway during bulblet initiation (**Figure 6A**). Without 2,4-D treatment, all screened DETs encoding Sucrose Synthase (SUS) (16 in *Lbg* and 17 in *Lb*) were significantly upregulated (*p* < 0.001) at 1 DAT or 8 DAT (**Figures 6B, C**). One Cell Wall Invertase (CWIN) in *Lbg* (Isoform 29531) were upregulated at 1 DAT and then downregulated, and the expression level of four CWINs (Isoform 29531, 28761 in *Lbg* and Isoform 39080, 34537 in *Lb*) were significantly increased (*p* < 0.001) in the 2,4-D-treated group (**Figures 6B, C**). Correspondingly, the content of glucose, *one of the* hexose hydrolytic *products of sucrose*, in the scales was significantly higher (*p* < 0.05) in the 2,4-D treated group than that in the control group in both *Lbg* and *Lb* (**Supplementary Figure S4**). Three Trehalose 6-Phosphate Synthases (TPSs) in *Lbg* (Isoform 36005, 19880 and 20796) and five TPSs in *Lb* (Isoform 24997, 32954, 34599, 24963 and 43346) were significantly upregulated (*p* < 0.001) at 1 DAT, 8 DAT or 14 DAT (**Figures 6B, C**). Notably, the expression patterns of some Sucrose Transporters (SUTs) and SWEET Sucrose-Efflux Transporters (SWEETs) were strongly affected by 2,4-D application. All screened differentially expressed SUTs (two in *Lbg* and two in *Lb*) were significantly upregulated (*p* < 0.001) at 1 DAT, among them, the upregulation of one SUT in *Lbg* (Isoform 31091) and two SUTs (Isoform 36404 and 36445) in *Lb* was significantly enhanced (*p* < 0.001) by 2,4-D (**Figures 6B, C**). In addition, there was no significant change in the expression level of three SWEETs in *Lbg* (Isoform 42147, 46206 and 42002) and one in *Lb* (Isoform 48829) without 2,4-D during bulblet initiation, but these SWEETs significantly upregulated (*p* < 0.001) at 8 DAT with 2,4-D application (**Figures 6B, C**).

Several genes encoding key enzymes involved in starch synthesis were upregulated during bulblet initiation. All screened DETs encoding large subunit of AGPase (AGPL), Granule-Bound Starch Synthase (GBSS), and Starch Synthase (SS) were significantly upregulated (*p* < 0.001) at 1 DAT without 2,4-D, but some of these upregulations were inhibited (Isoform 33352 in *Lbg*), attenuated (Isoform 33521, 37101, 19461, 29525, 30322 in *Lbg* and Isoform 44094 in *Lb*) or delayed (Isoform 28814 in *Lbg* and Isoform 32313, 32089, 30970, 44094, 35250, 46813, 30332 in *Lb*) in the 2,4-D-treated group (**Figures 6B, C**), which indicated that 2,4-D suppressed starch synthesis during bulblet initiation. On the contrary, the expression levels of some Amylases (AMYs) (Isoform 20315, 20650 in *Lbg* and Isoform 43835, 44457 in *Lb*) and β-Amylases (BAMs) (Isoform 29770, 29864 in *Lbg*), which were involved in starch degradation, were significantly upregulated (*p* < 0.001) by 2,4-D (**Figures 6B, C**), indicating that 2,4-D promoted starch degradation. In *Lbg*, the starch content of scales was significantly decreased (*p* < 0.05) by 2,4-D at 1 DAT, 8 DAT and 14 DAT (**Supplementary Figure S4**). Similar decreases were also observed in *Lb*, but there were no significant differences (*p* < 0.05) (**Supplementary Figure S4**). In addition, we identified five significantly upregulated Starch Branching Enzymes (SBEs) (*p* < 0.001) in *Lbg* (Isoform 22356, 21451, 22077, 26194 and 22461) but not in *Lb* (**Figures 6B, C**).

### 3.7 Identification of potential regulators of *in vitro* bulblet initiation

**Figure 7:**
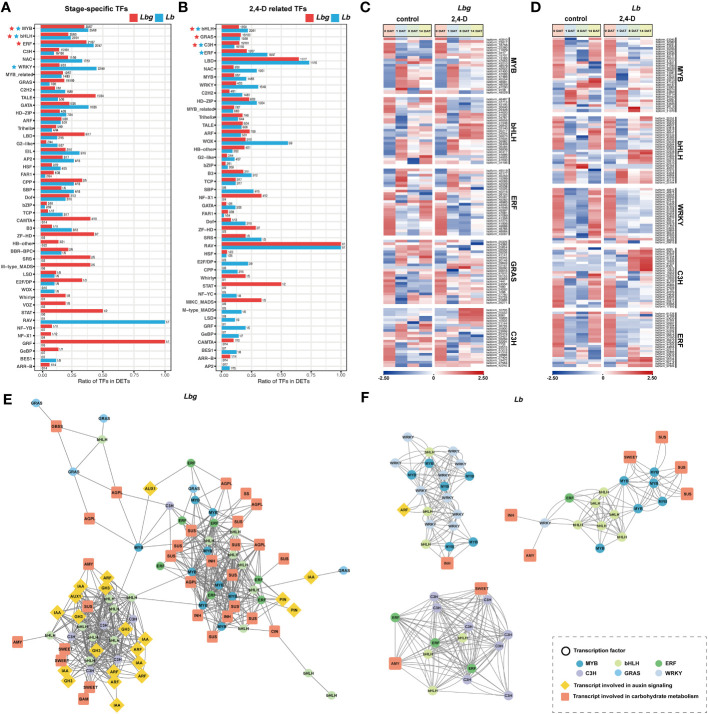
**(A)** TF families identified from the stage-specific DETs. **(B)** TF families identified from the 2,4-D-related DETs. The TF families in **(A)** and **(B)** rank according to the number of differentially expressed TFs they contained. **(C)** Express patterns of differentially expressed TFs belonging to MYB, bHLH, ERF, GRAS and C3H families in *Lbg*. **(D)** Express patterns of differentially expressed TFs belonging to MYB, bHLH, WRKY, C3H and ERF families in *Lb*. **(E, F)** Correlation analysis of DETs involved in auxin signaling and carbohydrate metabolism, and differentially expressed TFs in *Lbg* **(E)** and *Lb* **(F)**. The correlation analysis was conducted with Pearson’s two-tailed test. DETs with significant correlations (|r| ≥ 0.8. r, Pearson correlation coefficient) were linked. stering analysis of reference transcripts of *Lbg* **(A)** and *Lb* **(B)**.

In total, we identified 1209 and 1363 TFs from stage-specific DETs and 2,4-D-related DETs in *Lbg* and *Lb*, respectively. In stage-specific TFs, the number of MYB, bHLH and ERF ranked in the top three in *Lbg*, and MYB, bHLH and WRKY ranked in the top three in *Lb* (**Figure 7A**). In 2,4-D-related TFs, the three most were bHLH, GRAS and C3H in *Lbg*, and bHLH, C3H and ERF in *Lb* (**Figure 7B**). Given these, the expression patterns of TFs belonging to these TF families were analyzed, including 25 bHLHs, 24 GRASs, 22 C3Hs, 21 MYBs and 25 ERFs in *Lbg*, and 28 bHLHs, 25 C3Hs, 23 ERFs, 31 MYBs and 23 WRKYs in *Lb* (**Figures 7C, D**).

Further, the correlation analysis (|r| ≥ 0.8. r, Pearson correlation coefficient) between these candidate TFs and DETs involved in auxin signaling and carbohydrate metabolism was conducted. In *Lbg*, we found six bHLHs, seven ERFs, eight MYBs and one GRAS were co-expressed with ten SUSs, two INHs, one CIN, three AGPLs and two PINs (**Figure 7E**). Eight bHLHs and five C3Hs were co-expressed with three SWEETs, two AMYs, one BAM, one SUS and one AUX1, six Aux/IAAs, five ARFs and five GH3s (**Figure 7E**). In *Lb*, the expression level of six MYBs and six bHLHs were co-expressed with three SUSs and one SWEET (**Figure 7F**). Eight C3Hs three ERFs and three bHLHs were co-expressed with an AMY and a SWEET (**Figure 7F**). Ten WRKYs, six MYBs, three bHLHs and one ARF were co-expressed (**Figure 7F**). Above all, the above-mentioned TFs are possibly involved in the regulation of auxin signaling and carbohydrate metabolism, thus regulate the process of *in vitro* bulblet initiation.

## 4 Discussion

### 4.1 High-quality full-length transcripts of lily during *in vitro* bulblet initiation were constructed by a hybrid sequencing strategy

Tissue culture is a main asexual reproduction method for many bulbous crops and has a significant advantage in promoting regeneration efficiency and shortening the breeding and propagation cycle (Bakhshaie et al., 2016). Adventitious bud initiation and bulblet formation are critical steps during micropropagation of bulbous flowers, especially for direct organogenesis via shoot induction, which depends heavily on efficient nutritional allocation and hormone regulation (Xu et al., 2020a; Ren et al., 2022). Although many reports have been published on the process of bulblet formation and development in the lily (De Klerk, 2012; Li et al., 2014; Wu et al., 2021), there are relatively few reports on the detailed mechanism of early bulblet initiation.

In the present study, comprehensive full-length transcriptomes of *Lbg* and *Lb* were obtained through PacBio Iso-seq together with Illumina short-read sequencing during *in vitro* bulblet initiation. To date, Illumina sequencing technology has been widely applied to explore the process of bulblet formation and development in lily (Li et al., 2014; Du et al., 2017; Yang et al., 2017; Lazare et al., 2019). Recently, a full-length transcriptome of *Lilium* Oriental Hybrids ‘Sorbonne’ was generated (Li et al., 2022). Here, we conducted construct two high-quality full-length reference transcriptomes (*Lbg* and *Lb*). The N50 of transcripts of *Lbg* and *Lb* was 5,422 bp and 5,199 bp, respectively, and the mean length of transcripts of *Lbg* and *Lb* were 3,108 bp and 2,965 bp, respectively (**Supplementary Table S1**). These data contribute to further studies on molecular mechanisms of bulblet formation and other biological process of lily.

### 4.2 Exogenous 2,4-D application promote *in vitro* bulblet initiation by enhancing auxin signaling

Recent studies have indicated that auxin contributes to bulblet initiation in several bulbous flowers (Yang et al., 2017; Xu et al., 2020a). The content of IAA, the main naturally occurring auxin, increased consistently during the process of bulblet initiation and development in *Lycoris*, and exogenous IAA improved bulblet growth (Zhao, 2012; Xu et al., 2020a). In the present study, 1 mg L^-1^ 2,4-D significantly increase the regeneration rate of *in vitro* bulblet in *Lbg* and *Lb* (**Figure 5A**). Generally, auxin binds to TIR1 nuclear receptors, and then the auxin signal is modulated by the quantitative and qualitative responses of the Aux/IAAs and ARFs (Chapman and Estelle, 2009; Hayashi, 2012). ARFs are crucial regulators involved in the auxin signaling pathway, and then induce three major families: SAUR, GH3 and Aux/IAA genes (Guilfoyle and Hagen, 2007; Hayashi, 2012). In a previous study, differentially expressed TIRs, ARFs, and SAURs were identified during bulblet in *Lycoris* through transcriptome analysis (Xu et al., 2020a). Here, we found that 2,4-D affected the expression of genes involved in auxin signaling, thus promoted *in vitro* bulblet initiation. The obvious downregulations were found in many differentially expressed TIR1s at 1 DAT in both *Lbg* and *Lb*, which could be enhanced by 2,4-D application (**Figures 5B, C**). In addition, many ARFs, SAUR, GH3 and Aux/IAAs, which were not apparently responsive in the control group during bulblet initiation, were significantly upregulated in the 2,4-D-treated group (**Figures 5B, C**). Particularly, three ARFs in *Lbg* were significantly upregulated at 8 DAT in the control group (**Figure 5B**). Taken together, we proposed ARFs as key response factors during *in vitro* bulblet initiation in lily.

The distribution of auxin in cells depends largely on auxin transport, especially auxin efflux, which is directed by the polar subcellular localization of the PIN1 auxin efflux transporter in the plasma membrane (Vanneste and Friml, 2009; Hayashi, 2012). The expression and gradual polarization of PINs induced by auxin promote the formation of new vascular strands originating from the position of auxin application (Sauer et al., 2006). Three *PIN* genes were significantly upregulated during bulblet initiation in *Lycoris* (Xu et al., 2020a). In our study, all differentially expressed PINs were upregulated by 2,4-D in both *Lbg* and *Lb* (**Figures 5B, C**), indicating that 2,4-D might promote auxin transportation to promote bulblet initiation.

### 4.3 Starch and sucrose metabolism are crucial processes during *in vitro* bulblet initiation

The process of bulblet initiation involves carbohydrate transport from the source to the sink. The starch storage in the mother scales and exogenous carbon supply can be considered as the carbon source, and the basal of the mother scale, where the bulblets initiate, act as the sink tissue. Sucrose is unloaded from the phloem into sink cells either apoplasmically or symplasmically, then utilized to produce energy for cellular process (Ruan, 2014; **Figure 6A**).

Starch accounts for approximately 70% of the dry weight of lily bulbs (Wu et al., 2021). Starch synthesis is considered to be a crucial pathway for bulblet initiation. Several studies have indicated that whether a meristem can produce scale primordia depends on its capacity to accumulate starch (Bourque et al., 1987; Wu et al., 2021). In *Lycoris*, abscisic acid (ABA) upregulated the expression level of *LrSS1*, *LrSS2*, and *LrGBSS1* genes, which could enhance carbohydrate accumulation in the bulblets, thus promoted their development (Xu et al., 2020b). Similarly, starch synthesis was positively correlated with bulbil formation in *Lilium lancifolium* with upregulation of *AGPL*, *SS*, *GBSS* and *SBE* (Yang et al., 2017). In the present study, all screened DETs encoding AGPL, GBSS, and SS were upregulated at 1 DAT or 8 DAT without 2,4-D in both *Lbg* and *Lb* (**Figures 6B, C**), indicating the enhancement of starch synthesis process. Especially, five significantly upregulated SBEs were identified in *Lbg* (**Figures 6B, C**), indicating that *Lbg* might have a stronger ability of starch accumulation for bulblet initiation than *Lb*. During bulblet formation, starch is degraded in the mother scales and synthesized at the bulblet regeneration site and in the newly formed bulblets. In *Lilium*, the enzymes involved in starch synthetic direction, such as AGPase, GBSS, SS, and SBE, showed a decreasing trend in mother scales but an increasing trend in bulblets during bulblet formation (Li et al., 2014; Wu et al., 2021). Moreover, starch content in basal scales and basal plates of *Lycoris* (the major sites of bulblet regeneration) showed a rapid decline during bulblet initiation in the efficient bulblet regeneration system (Ren et al., 2021). Here, we found in the 2,4-D-treated group, the more efficient group for *in vitro* bulblet initiation, key enzymes involved in starch synthesis (AGPL, SS and GBSS) were downregulated, while key enzymes involved in starch degradation (AMY and BAM) were upregulated compared to the control group (**Figures 6B, C**). In addition, 2,4-D reduced the starch content in the scales during bulblet initiation (**Supplementary Figure S4**). Taken together, we suggested that 2,4-D accelerate the starch degradation process to increase carbon supply for newly bulblet initiation.

Soluble sugars in mother scales were transported into the region where bulblets were initiated to supply the follow-up bulblet development (Xu et al., 2020a). Sucrose is the dominant transport form of sugars in higher plants (Lalonde et al., 1999; Rolland et al., 2006). SUS and CWIN are considered the most important sucrose hydrolases involved in the sucrose unloading pathway (Ruan, 2014; **Figure 6A**). SUS contribute to starch synthesis and accumulation, functioning during the later bulblet initiation and development (Yang et al., 2017; Wu et al., 2021). In *Lilium*, *SUS*s and *INV*s were both highly expressed in the mother scales and bulblets during bulblet emergence and swelling (Li et al., 2014). Similarly, *SUS*s and *INV*s were greatly upregulated accompanied by a decrease in sucrose content in mother scales during bulblet initiation in *Lycoris* (Xu et al., 2020a). In this study, 16 SUSs in *Lbg* and 17 SUSs in *Lb* were upregulated at 1 DAT or 8 DAT, and one CWIN in *Lbg* and two CWINs in *Lb* were upregulated at 1 DAT and then downregulated (**Figures 6B, C**). For example, *SUS* and *CWIN* often presented an opposite expression pattern during bulblet initiation, and this change was considered to be a possible sign of the transition from bulblet initiation to development (Ren et al., 2021; Wu et al., 2021). In particular, *CWINs* were highly expressed during the early bulblet initiation stage and produced glucose, which might act as sugar signaling rather than carbon resources (Wu et al., 2021). In *Lycoris*, the more highly *LsCWIN2* was expressed, the more bulblets were produced (Ren et al., 2021). Here, the expression levels of CWINs in *Lbg* and *Lb* were significantly increased by 2,4-D, showing a possible role of CWINs in increasing bulblet regeneration rate. Recent study showed that *LbgCWIN1* significantly upregulated endogenous starch was degraded during *in vitro* bulblet initiation in lily (Gao et al., 2023), indicating that *CWIN* can be selected as a candidate gene subsequent function verification.

Interestingly, the application of 2,4-D in the medium for bulblet induction had significant effects on the expression of genes involved in carbohydrate metabolism, especially SUTs and SWEETs (**Figure 6A**). SWEETs probably mediate sucrose efflux from SE/CC to apoplasm, and then sucrose can be taken up by SUTs, which are key steps proceeding phloem unloading (Ruan, 2014). The upregulation of one SUT in *Lbg* and two SUTs in *Lb* was significantly enhanced by 2,4-D at 1 DAT (**Figures 6B, C**). Three SWEETs in *Lbg* and one SWEET had no significant expression change in *Lb* without 2,4-D during bulblet initiation, but significantly upregulated at 8 DAT and 14 DAT with 2,4-D application (**Figures 6B, C**). Thus, 2,4-D might facilitate *in vitro* bulblet initiation mainly through promoting sucrose unloading from the SE/CC to the sink cells, and *SWEETs* and *SUTs* can be considered as good candidates for future functional studies.

### 4.4 Candidate TFs might be involved in the regulation of *in vitro* bulblet initiation

Although carbohydrate metabolism and the auxin signaling pathway have been respectively demonstrated to participate in bulblet initiation, their cooperative function during this process has not yet been reported. Here, we found that in *Lbg*, three SWEETs, two AMYs, one BAM and one SUS were co-expressed with one AUX1, six Aux/IAAs, five ARFs and five GH3s, and ten SUSs, two INHs, one CIN and three AGPLs were co-expressed with two PINs (**Figure 7E**), indicating the possible coregulation by carbohydrate metabolism and auxin signaling of *in vitro* bulblet initiation in lily. A recent study showed that bHLH, bZIP, WRKY, TCP, MYB, YABBY, NAC, C2H2 were identified to be involved in bulbil induction of ‘Sorbonne’ lily by coexpression analysis (Li et al., 2022). In *Lycoris*, coexpression analysis revealed that transcripts encoding WOX14, MYB117, and ULT1 coexpressed with *LsCWIN2*, and transcripts encoding ERFs were coexpressed with *LsSUS4* during bulb vegetative production (Ren et al., 2022). In the present study, MYB, bHLH, ERF, C3H, GRAS, WRKY families were identified to be candidate regulators of *in vitro* bulblet initiation. Among them, several TFs might be involved in the regulation the expression of key enzymes in carbohydrate metabolism during *in vitro* bulblet initiation in both *Lbg* and *Lb*, for example, C3Hs and bHLHs were co-expressed with AMYs and SWEETs, and some MYBs and bHLHs were co-expressed with SUSs in both *Lbg* and in *Lb* (**Figures 7E, F**). The above results may assist in the understanding of molecular mechanism of lily bulblet initiation.

## Data availability statement

The data presented in the study are deposited in the GenBank repository, accession number PRJNA933000.

## Author contributions

LZ, YW and ZMR designed the experiment. LZ, CG, YCX, YL and XX carried out the experiment. CG and LC analyzed the data. CG drafted the manuscript. ZR, YW and YPX revised the manuscript. All authors contributed to the article and approved the submitted version.

## Supplementary material

Supplementary Table 1 Statistical summary of the reference transcriptome.

Supplementary Table 2 Primers of selected transcripts for quantitative real-time PCR validation.
