# Supplementary figures and images for: Full-length transcriptome analysis revealed that 2,4-dichlorophenoxyacetic acid promoted in vitro bulblet initiation in lily by affecting carbohydrate metabolism and auxin signaling
Source: Front Plant Sci. 2023 Sep 20;14:1236315. doi: 10.3389/fpls.2023.1236315 (PMC10548195; doi:10.3389/fpls.2023.1236315)

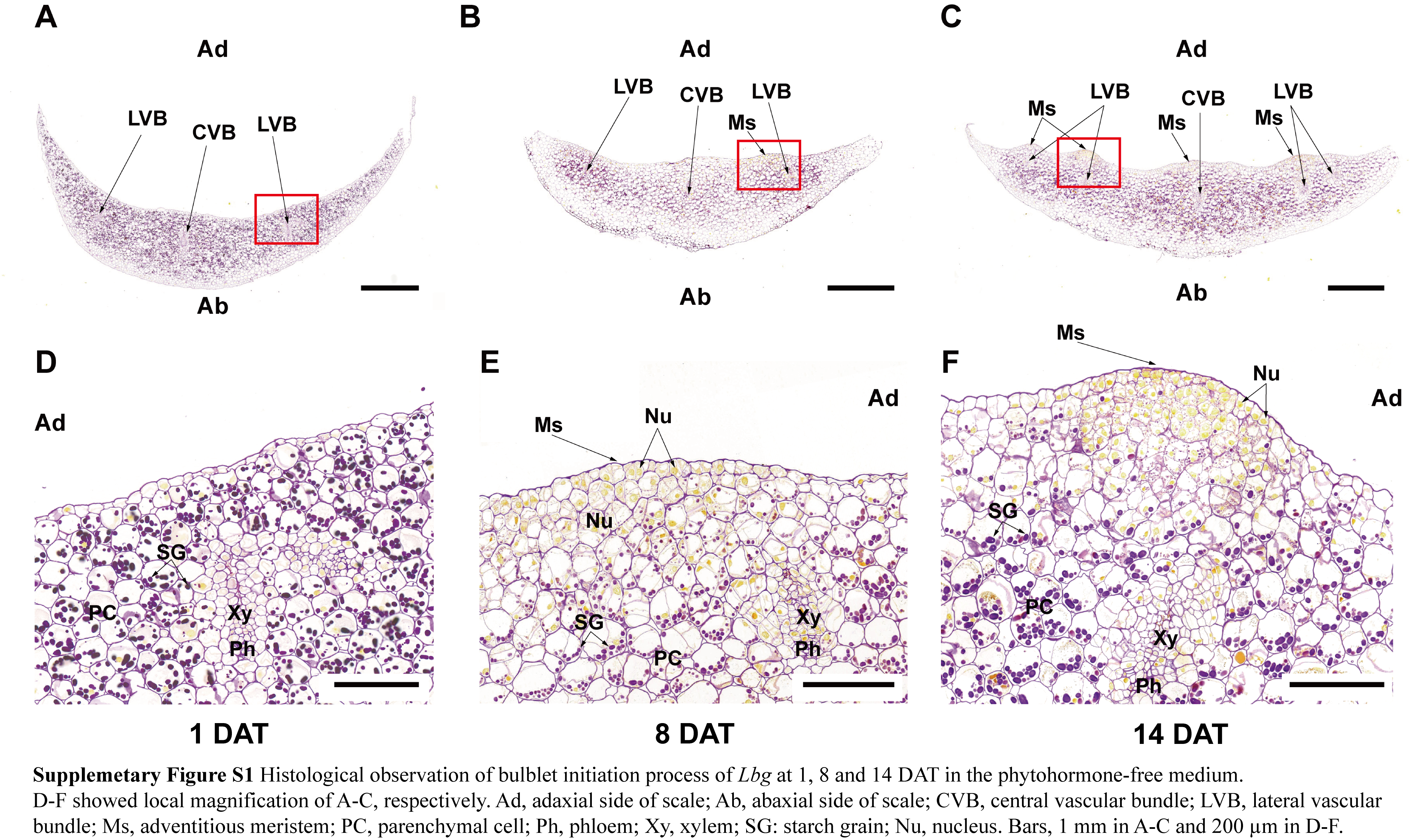

Supplement: Supplementary file 1 [file Image_1.tif]

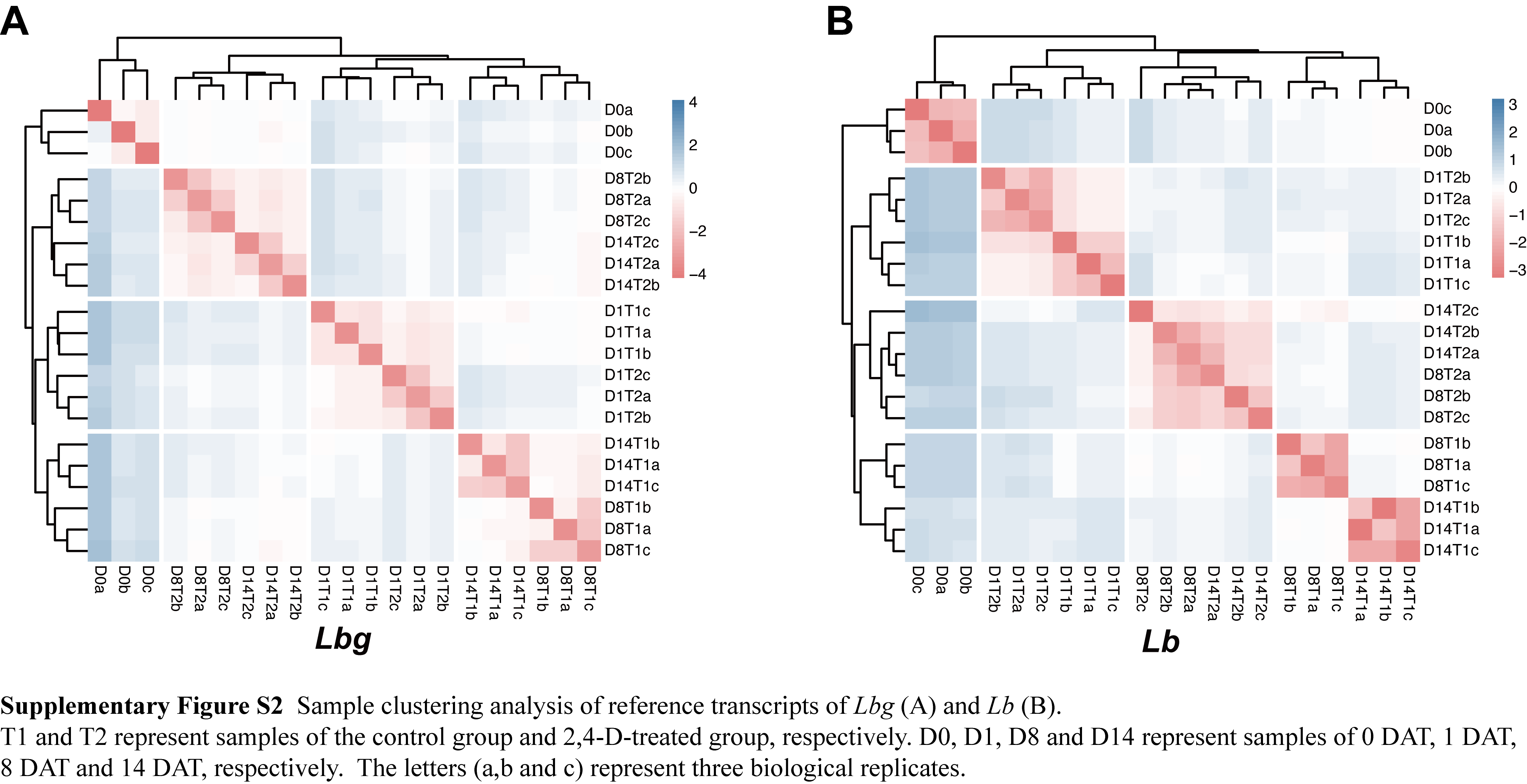

Supplement: Supplementary file 2 [file Image_2.tif]

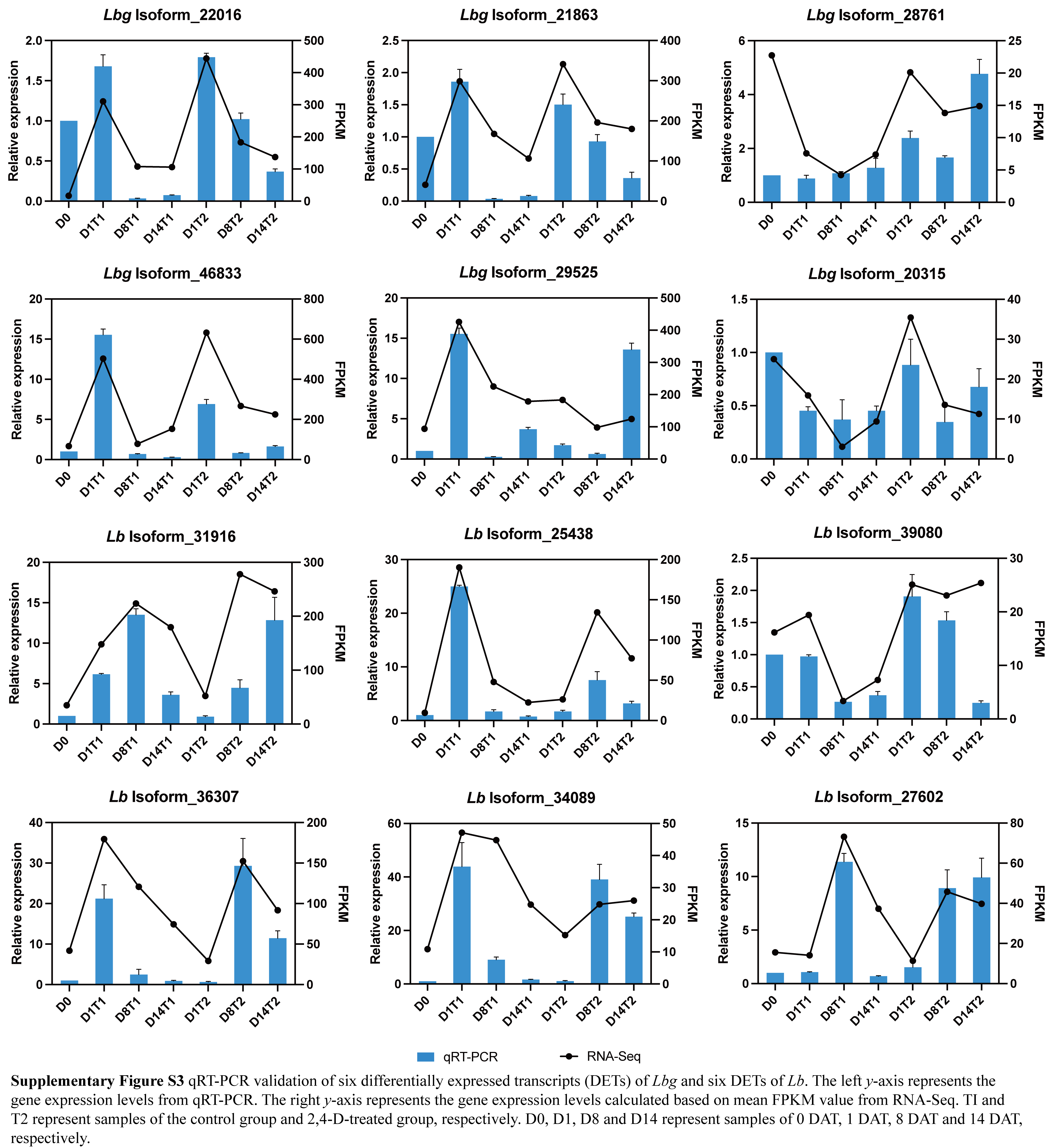

Supplement: Supplementary file 3 [file Image_3.tif]

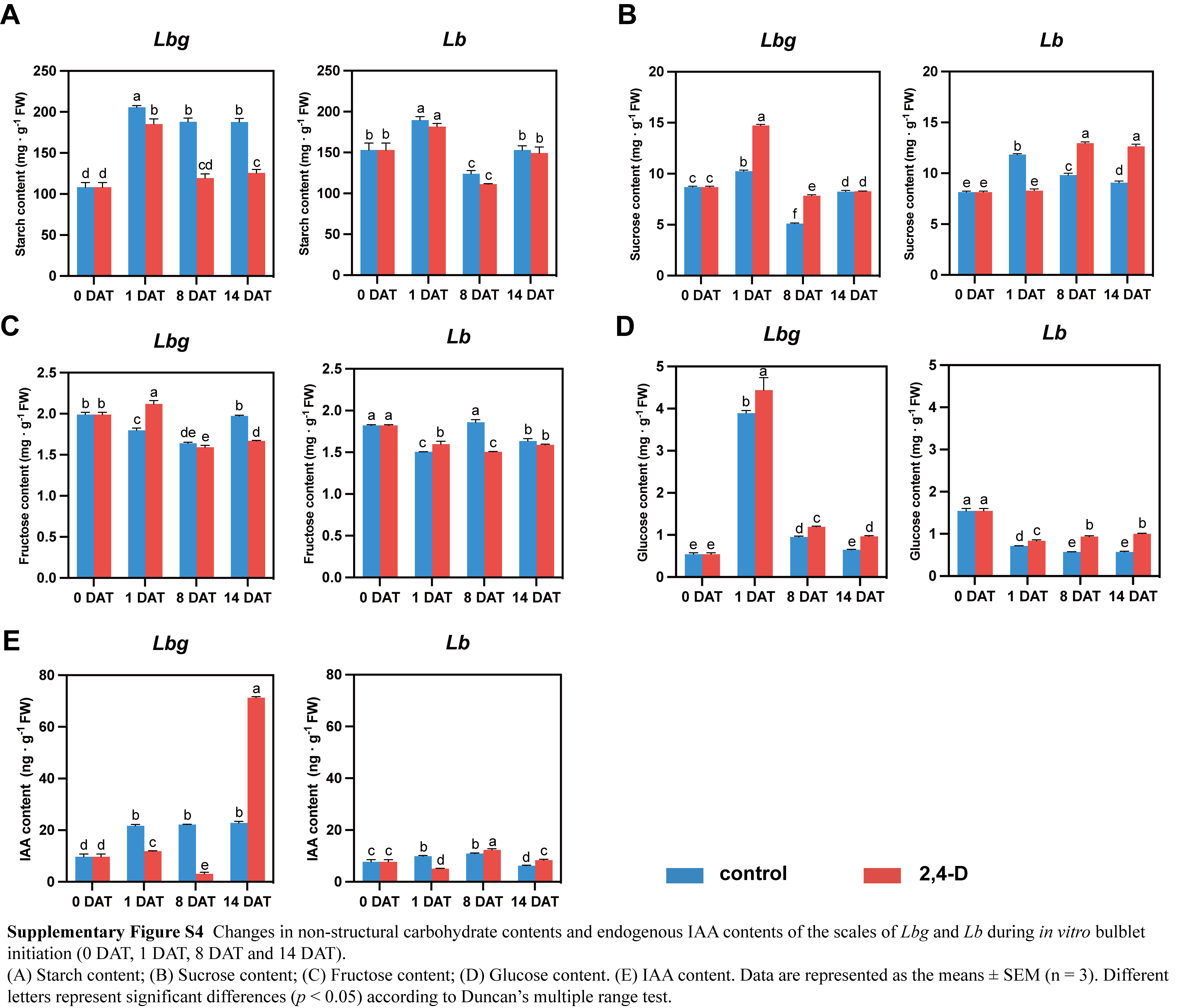

Supplement: Supplementary file 4 [file Image_4.tif]
